# Tumor and Organ Dose Estimates from ^177^Lu-PSMA-617 Therapy in Patients with Metastatic Castration-Resistant Prostate Cancer: Results from the RESIST-PC Phase 2 Trial

**DOI:** 10.2967/jnumed.126.272574

**Published:** 2026-10

**Authors:** Zachary Ells, Catherine Meyer, Karam Masri, Marc Ryhiner, David Sennung, Koichiro Kimura, David Mirando, Izabela Tworowska, Rouzbeh Esfandiari, Martin S. Allen-Auerbach, Michael Lassmann, Ken Herrmann, Matthias Eiber, Wolfgang P. Fendler, Ebrahim Delpassand, Johannes Czernin, Magnus Dahlbom, Jeremie Calais

**Affiliations:** 1 Department of Nuclear Medicine and Theranostics, Ahmanson Translational Theranostics Division, David Geffen School of Medicine, UCLA, Los Angeles, California;; 2 Department of Nuclear Medicine, Inselspital, University of Bern, Bern, Switzerland;; 3 MIM Software, Beachwood, Ohio;; 4 RadioMedix Inc., Houston, Texas;; 5 Excel Diagnostics & Nuclear Oncology Center, Houston, Texas;; 6 Department of Nuclear Medicine, Würzburg University Hospital, Würzburg, Germany;; 7 University of Essen, Essen, Germany;; 8 Department of Nuclear Medicine, West German Cancer Center, DKTK and NCT site, University Hospital Essen, Essen, Germany;; 9 Technical University of Munich, Munich, Germany; and; 10 Bavarian Cancer Research Center, Erlangen, Germany

**Keywords:** ^177^Lu-PSMA, radiopharmaceutical therapy, prostate cancer, dosimetry

## Abstract

This is a dosimetry analysis of the prospective randomized phase 2 trial RESIST-PC which evaluated the efficacy and safety of 2 injected activity regimens of [^177^Lu]Lu-PSMA-617 in patients with progressive metastatic castration-resistant prostate cancer. **Methods:** This biinstitutional study randomized patients with metastatic castration-resistant prostate cancer to receive either 6.0 or 7.4 GBq of [^177^Lu]Lu-PSMA-617 per cycle for up to 4 cycles (1:1 randomization). The γ-images were obtained at the first cycle using a hybrid protocol (planar + SPECT). Whole-body planar scintigraphy images were acquired at 4, 24, 48, and 72 h (optionally at 168 h), and a quantitative SPECT/CT was performed at 24 h postinjection. Absorbed doses (ADs) were calculated for the kidneys, salivary glands, liver, and tumors. Planar time–activity curves were derived on a per-region basis from counts in the serial scans, with exponential curve fit selection based on Akaike information criterion. Quantitative SPECT data per region were scaled by the planar time–activity data and then convolved with a voxel-*S* value dose kernel to yield AD estimates. No partial volume corrections were applied. Adverse events and efficacy data were correlated to the calculated AD. **Results:** A total of 48 of 64 patients (16 treated with 6.0 GBq vs. 32 in the 7.4-GBq arm) had complete imaging datasets and were analyzed. The mean kidney, submandibular, parotid, and liver ADs were 0.29 ± 0.11, 0.23 ± 0.18, 0.26 ± 0.15, and 0.11 ± 0.09 Gy/GBq, respectively. There was no significant difference in organ or tumor ADs between arms. In total, 358 lesions were included in the analysis (median, 7/patient [range, 0–18/patient]). The mean AD to bone lesions (*n* = 304) was 1.90 ± 1.83 and 1.64 ± 1.77 Gy/GBq for the 6.0-GBq (*n* = 121) and 7.4-GBq (*n* = 183) groups, respectively. For lymph node lesions (*n* = 35), mean ADs were 5.61 ± 3.93 Gy/GBq (*n* = 22) and 1.79 ± 1.96 Gy/GBq (*n* = 13) for the 6.0- and 7.4-GBq groups, respectively. Only the 7.4-GBq cohort presented liver lesions; mean AD was 1.59 ± 1.45 Gy/GBq (*n* = 19). A statistically significant inverse correlation was observed between prostate-specific antigen response and tumor AD (ρ = –0.404, *P* = 0.006). **Conclusion:** In the RESIST-PC prospective phase 2 trial, dosimetry analyses using a hybrid imaging protocol revealed organ ADs within tolerable ranges, and tumor ADs varied widely on the inter- and intrapatient level regardless of injected activity.

## Visual Abstract

**Figure:**
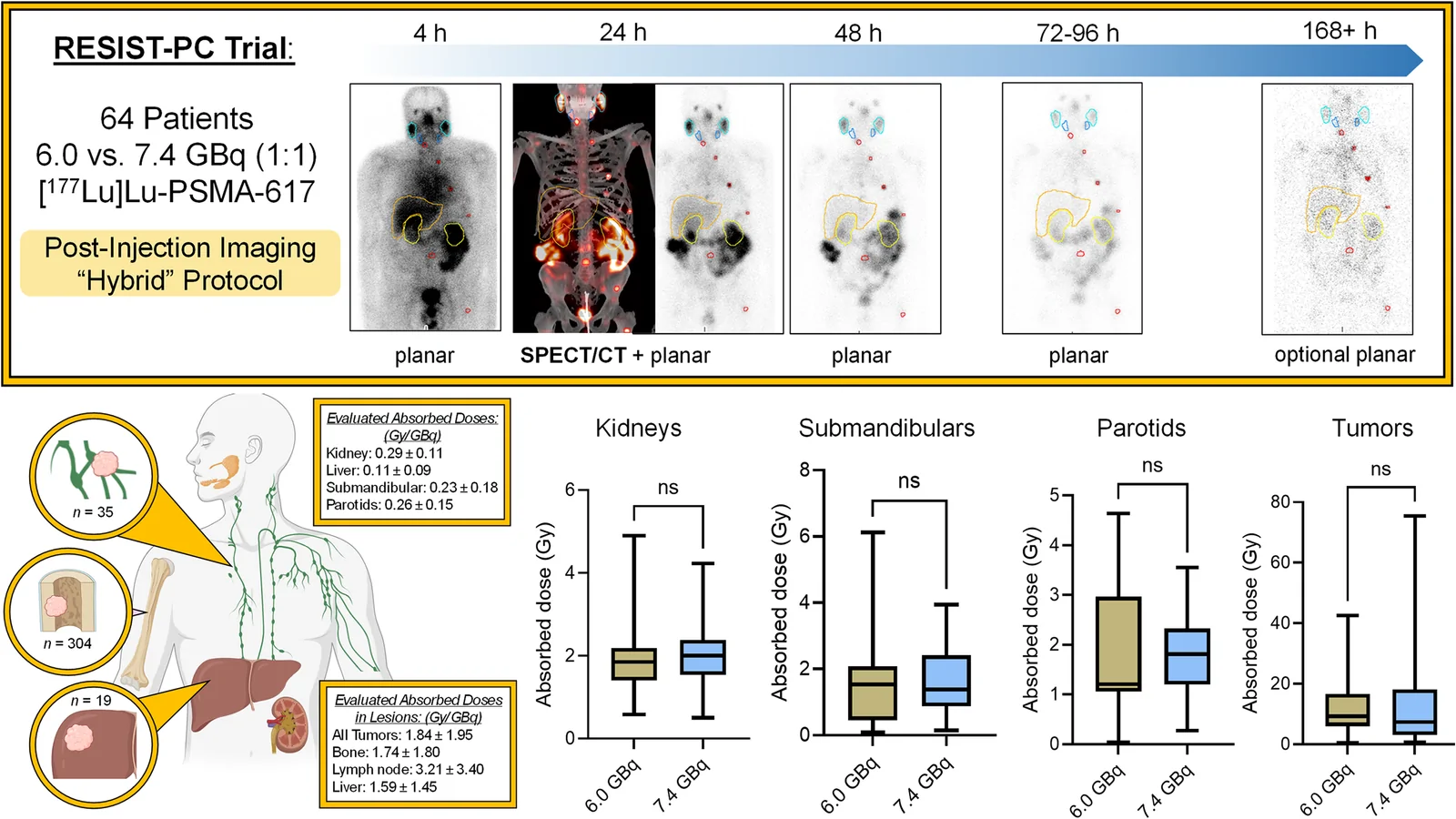

Prostate-specific membrane antigen (PSMA) is expressed at high levels in prostate cancers (1) and is an established target for diagnostic imaging (2–4) and therapeutic delivery (5–7). The randomized phase 3 VISION trial (NCT03511664) (8) demonstrated the validity of PSMA as a target and ^177^Lu as a useful isotope by significantly increasing overall survival (OS) and progression-free survival (PFS) versus standard of care in the treatment of metastatic castration-resistant prostate cancer (mCRPC). This led to the approval of [^177^Lu]Lu-PSMA-617 (Pluvicto; Novartis) by the Food and Drug Administration and the European Medicines Agency (9).

The RESIST-PC trial (PSMA-directed endoradiotherapy of castration-resistant prostate cancer; NCT03042312) was designed in 2017 to assess the efficacy and safety of 2 different injected activity regimens of [^177^Lu]Lu-PSMA-617 to treat mCRPC: 6.0 versus 7.4 GBq. A previous publication summarizes the safety results, but briefly, both activity regimens were well tolerated, with side effects easily managed by standard medical support (10). The Food and Drug Association required that the trial include a renal safety assessment in the form of kidney absorbed dose (AD) estimates during the first cycle of [^177^Lu]Lu-PSMA-617, using a multiple-time-point hybrid imaging protocol (planar + SPECT) for safety purposes. Beyond possible renal toxicity, red marrow and salivary gland toxicities (including xerostomia) are possible dose-limiting factors in the administration of small-molecule ^177^Lu-PSMA–based radiopharmaceutical therapies (11–13). Dose–response relationships relating AD to adverse events and clinical outcomes remain a key open question in the field (14).

The goal of this retrospective analysis was to compare the ADs from the 2 injected activity regimens of [^177^Lu]Lu-PSMA-617 (6.0 and 7.4 GBq) in organs at risk and lesions, with their association to treatment response and adverse events.

## MATERIALS AND METHODS

### Study Design and Patient Population

RESIST-PC was a bicentric prospective phase 2 trial conducted at the UCLA and Excel Diagnostics and Nuclear Oncology Center. Patients with advanced mCRPC progressive after or during androgen receptor–directed therapy (abiraterone or enzalutamide) or chemotherapy-naïve or chemotherapy-treated were eligible. No prior radiopharmaceutical therapy was permitted. Patients were randomized 1:1 to receive up to 4 cycles of 6.0 or 7.4 GBq of [^177^Lu]Lu-PSMA-617 therapy every 8 wk. The study design, clinical efficacy, and safety outcomes were previously reported (10,15).

### Hybrid Imaging Protocol

Serial [^177^Lu]Lu-PSMA-617 γ-camera images were acquired at the first cycle of therapy using either a Siemens Symbia Intevo 6, Symbia T2, or Symbia T6 scanner (all equipped with 3/8 in. NaI crystals). Patients underwent whole-body scintigraphy imaging at 4 time points: 4, 24, 48, and 72–96 h, with an optional delayed scan 168–216 h postinjection. Planar images were acquired with a symmetric 20% energy window centered at 208 keV using a medium-energy collimator. The whole-body scan rate was 20 cm/min (matrix, 1024 × 256). Patients also underwent a single SPECT/CT acquired at 24 h postinjection (60 views/head; 15 s/view). SPECT acquisitions were followed by a low-dose CT acquisition for attenuation correction (parameters: 130 kV, 30 mAs, 5.0-mm slice thickness, pixel size 1.0 mm^2^). Scatter correction was applied using triple-energy windows (10% width upper and lower scatter windows around 208 keV).

### Quantitative SPECT Reconstruction

All SPECT images were reconstructed using MIM SPECTRA Quant (GE HealthCare’s MIM Software, version 7.3.5). Image datasets were reconstructed using an ordered-subsets expectation maximization algorithm with 48 total updates [48 iterations 1 subset for scans acquired on the Symbia Intevo 6 (*n* = 28) and Symbia T6 (*n* = 6) or 24 iterations 2 subsets for scans acquired on the Symbia T2 (*n* = 14)].

The ^177^Lu SPECT images were quantified using phantom-derived calibration factors, following the European Association of Nuclear Medicine’s dosimetry guidelines (16). The following image calibration factors (17) were independently determined and implemented into the reconstruction for the Symbia Intevo 6, Symbia T2, and Symbia T6 cameras: 20.2, 19.6, and 20.0 counts per second/MBq, respectively (procedural details are available in supplemental materials, available at http://jnm.snmjournals.org).

### Segmentation

Organs-at-risk segmentation was completed using a deep learning–assisted organ segmentation (Contour ProtégéAI; MIM Software) on the 24 h postinjection CT. To be included in the analysis, lesions must have been clearly identifiable on all time points of planar imaging. Additionally, lesions were excluded if there was an overlapping activity signal from other tissues on the planar scans. Total tumor burden was defined as the sum of all lesions meeting said criteria. Tumor segmentation on the 24 h postinjection SPECT/CT was completed using the automatic edge detection algorithm (PET Edge Plus; MIM Software) by an experienced nuclear medicine physician. Three different anatomic lesion locations were determined (bone, lymph node, and liver).

All 3-dimensional volumes of interest from the SPECT/CT were propagated onto the planar series as 2-dimensional regions of interest. The planar and SPECT scans were automatically coregistered to each other using rigid registration with manual alignment adjustment if necessary.

### AD Estimates

A commercial hybrid imaging dosimetry protocol was used to estimate ADs (dosimetry with 1 SPECT/CT and serial planar imaging; MIM SurePlan MRT, version 7.3.5). Organ time–activity data were defined on a per-region level on the basis of the geometric mean of the planar images. However, planar time–activity data for the kidneys was derived using only counts in the posterior views to limit overlapping counts from the liver and other gastrointestinal organs. Planar time–activity data for tumors were defined using counts from posterior, anterior, or geometric mean on an individual basis based on the lesion position (depth).

The planar time–activity data was then then fit using a mono- or biexponential model (Levenberg–Marquardt algorithm to optimize each fit using sum-squared errors and Akaike information criterion to select the best function) and integrated over time. The SPECT was converted to time-integrated activity by multiplying the ratio between the planar time-integrated activity curve and the 24-h planar time point data (matching 24 h SPECT) on a per-structure basis (CT-based). The resulting voxelized time-integrated activity map was then convolved with a voxel-*S* value ^177^Lu kernel to yield AD estimates. The average AD deposition over all selected lesions was determined. No partial-volume effect corrections were applied.

### Clinical Outcomes

All patient-reported and clinically determined adverse events documented in the RECIST-PC trial were reviewed and classified by 2 investigator physicians by consensus into 7 categories. These included toxicities related to bone marrow (e.g., anemia, thrombocytopenia, lymphopenia, etc.), salivary glands (e.g., xerostomia), kidneys (nephrotoxicity clinical parameter changes, for example, estimated glomerular filtration rate or creatinine), gastrointestinal tract (nausea, vomiting, diarrhea, constipation, etc.), or liver, fatigue, and pain. All adverse events considered for analysis with cycle 1 ADs were required to have onset before the second treatment cycle.

Additionally, efficacy data including prostate-specific antigen (PSA) PFS (PSA-PFS), OS, and best PSA response (largest decrease in PSA over treatment course reported) were collected.

### Statistical Analysis

A Mann–Whitney *t* test was used to compare the tumor volumes and ADs of the 2 injected activity cohorts after confirmed by a Shapiro–Wilks test for normality. Between the 2 injected activities, a *P* value of less than 0.05 was deemed statistically significant.

To evaluate correlation between calculated ADs, patient-reported adverse events [descriptive statistical data (e.g., none, mild, moderate, or severe)], and efficacy outcomes, the Spearman ρ test was used. It was a monotonic correlation analysis with a *P* value of less than 0.05 deemed statistically significant.

## RESULTS

Between July 2017 and July 2018, 64 patients with mCRPC were enrolled and received [^177^Lu]Lu-PSMA-617 therapy (30 patients treated with 6.0 GBq vs. 34 in the 7.4-GBq arm) (10,15). In total, 48 (75%) patients with complete imaging datasets (16 and 32 patients in the 6.0- and 7.4-GBq arms, respectively) were included in this analysis. The patient inclusion flowchart is shown in Figure 1.

**FIGURE 1.**
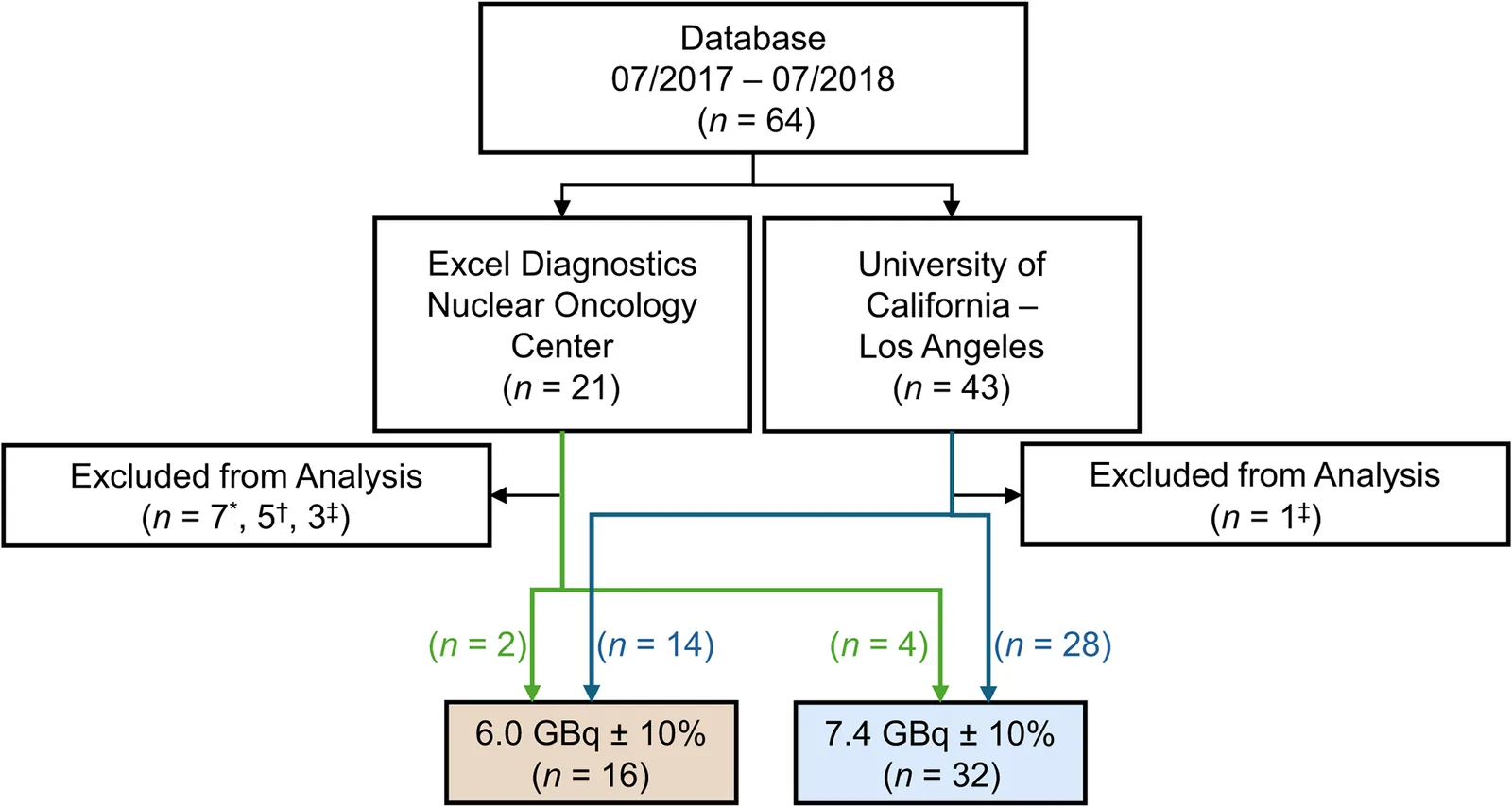
Patient inclusion in analysis. *Dataset was not available for analysis. ^†^Error in acquisition of dataset. ^‡^Error in registration between SPECT and CT.

### Imaging for Dosimetry

Of the 48 patients, 47 (98%) completed serial planar imaging at 4, 24, 48, and 72 h postinjection, with a quantitative SPECT acquired at 24 h (1 (2%) patient was scanned at 96 h instead of 72 h). Additionally, 7 of 48 patients (15%) completed the optional delayed time point planar scan after more than 168 h postinjection.

Figures 2 and 3 display the image series for patients with either 4 or 5 total planar scans, respectively, with each patient randomized to receive 6.0 and 7.4 GBq.

**FIGURE 2.**
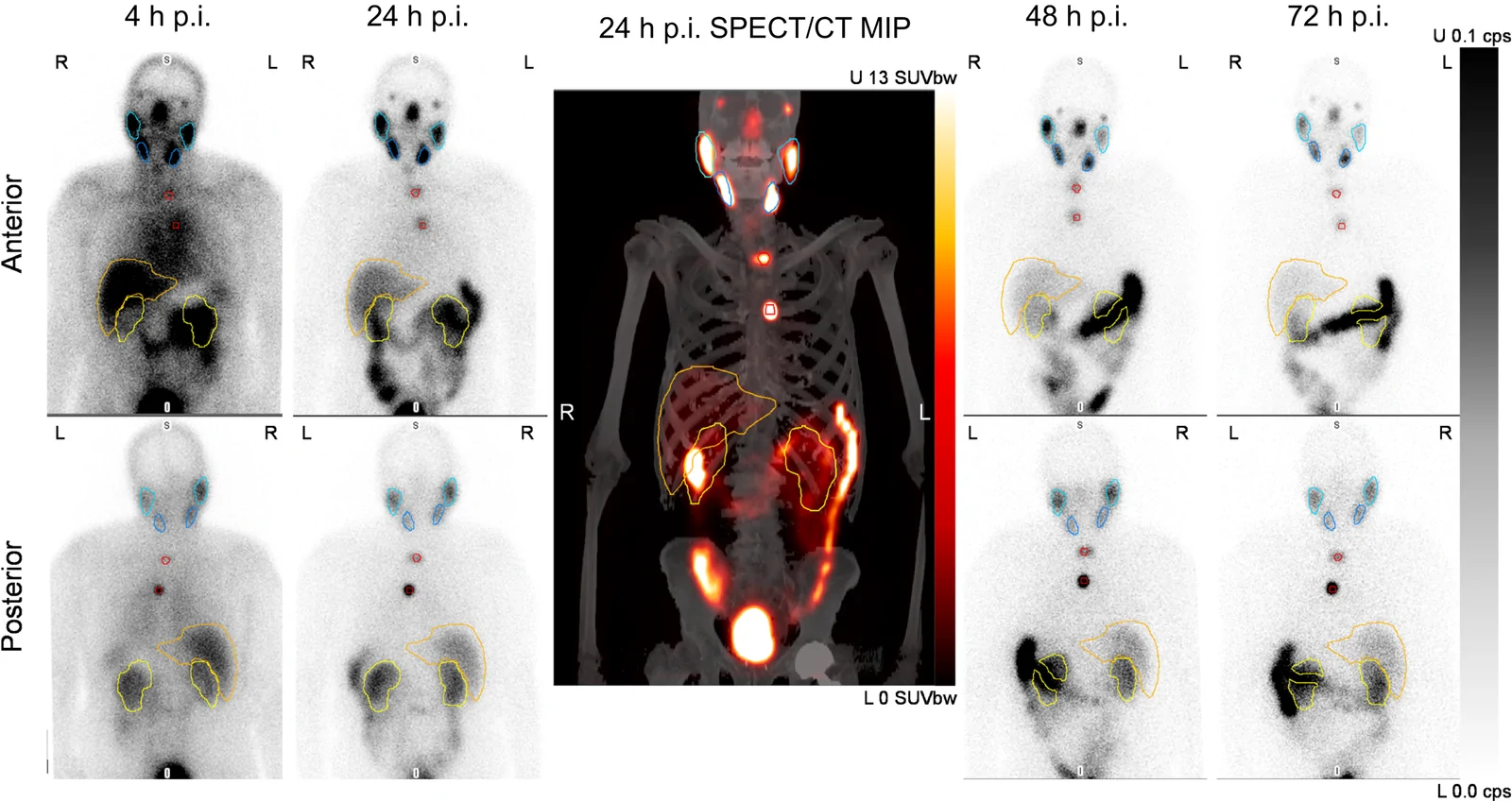
83-y-old patient randomized to 6.0-GBq cohort, with 4 imaging time points acquired postinjection. Two lesions (red) in sternum were visible on all time points and were included for analysis. For inferior lesion (volume, 2.0 mL; AD, 20.2 Gy), we used only posterior views, whereas for superior lesion (volume 2.8 mL, AD, 4.4 Gy), we used geometric mean. Note overlapping abdominal activity removed from kidney regions (yellow) on late time points (48 and 72 h). cps = counts per second; MIP = maximum-intensity projection; p.i. = postinjection; SUVbw = SUV normalized for body weight.

**FIGURE 3.**
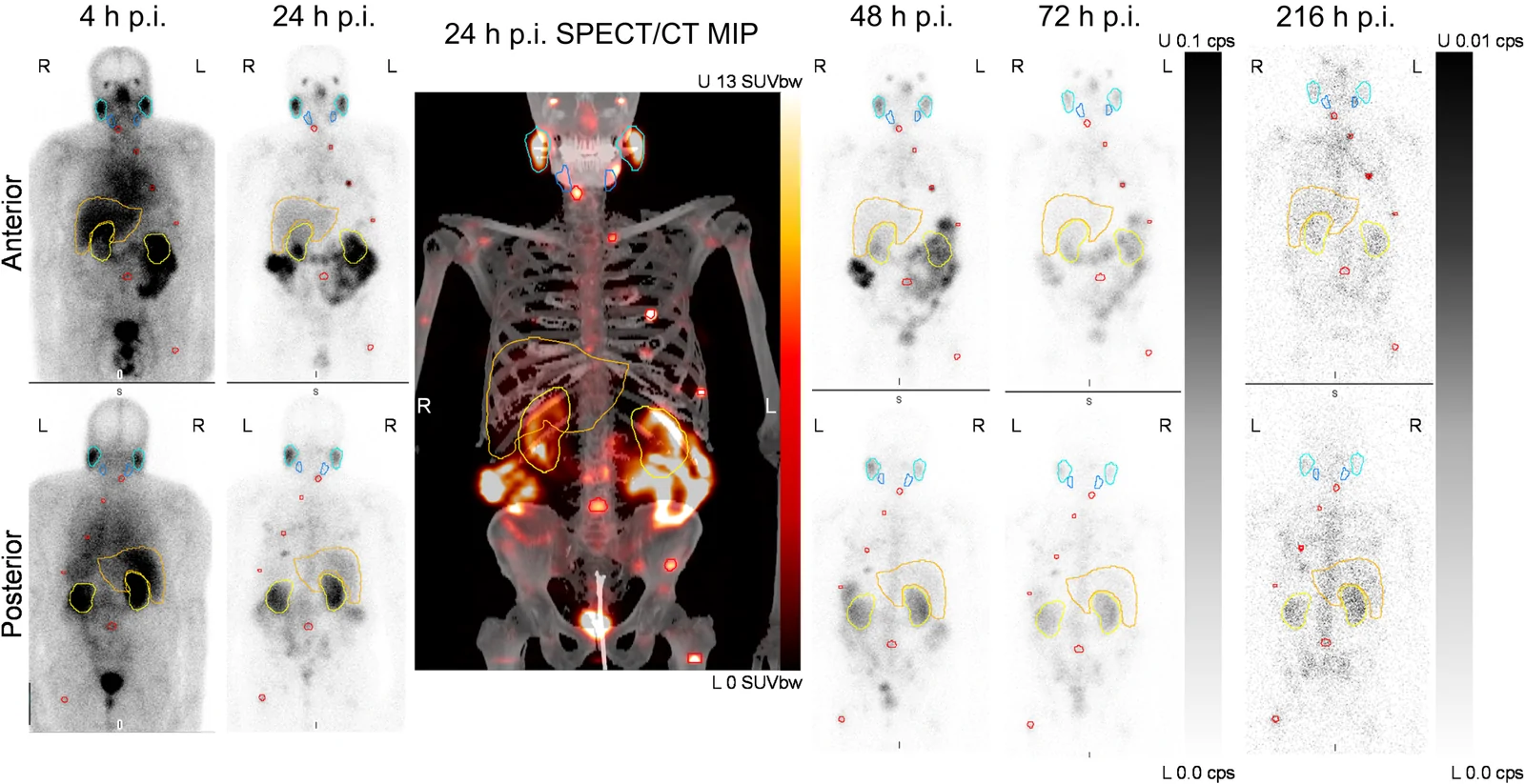
77-y-old patient randomized to 7.4-GBq cohort, with 5 imaging time points acquired postinjection. Six bone lesions (red) were visible on all time points and were included for analysis. From superior to inferior, lesion volume and ADs were as follows: 2.26 mL, 2.16 Gy; 0.72 mL, 1.71 Gy; 0.91 mL, 6.58 Gy; 0.85 mL, 2.48 Gy; 5.71 mL, 1.99 Gy; and 1.77 mL, 7.3 Gy. cps = counts per second; MIP = maximum-intensity projection; p.i. = postinjection; SUVbw = SUV normalized for body weight.

### Organ Dosimetry

AD estimates to the kidney, liver, submandibular glands, and parotid glands are summarized in Figure 4 and Table 1. The mean ± SD ADs for kidneys, submandibular glands, parotid glands, and liver for the whole cohort were 2.01 ± 0.81, 1.59 ± 1.17, 1.79 ± 0.96, and 0.77 ± 0.64 Gy, respectively. There was no significant difference in organ ADs between the 2 injection activity regimens for either the absolute AD or AD normalized by activity, as shown in Table 1.

**FIGURE 4.**
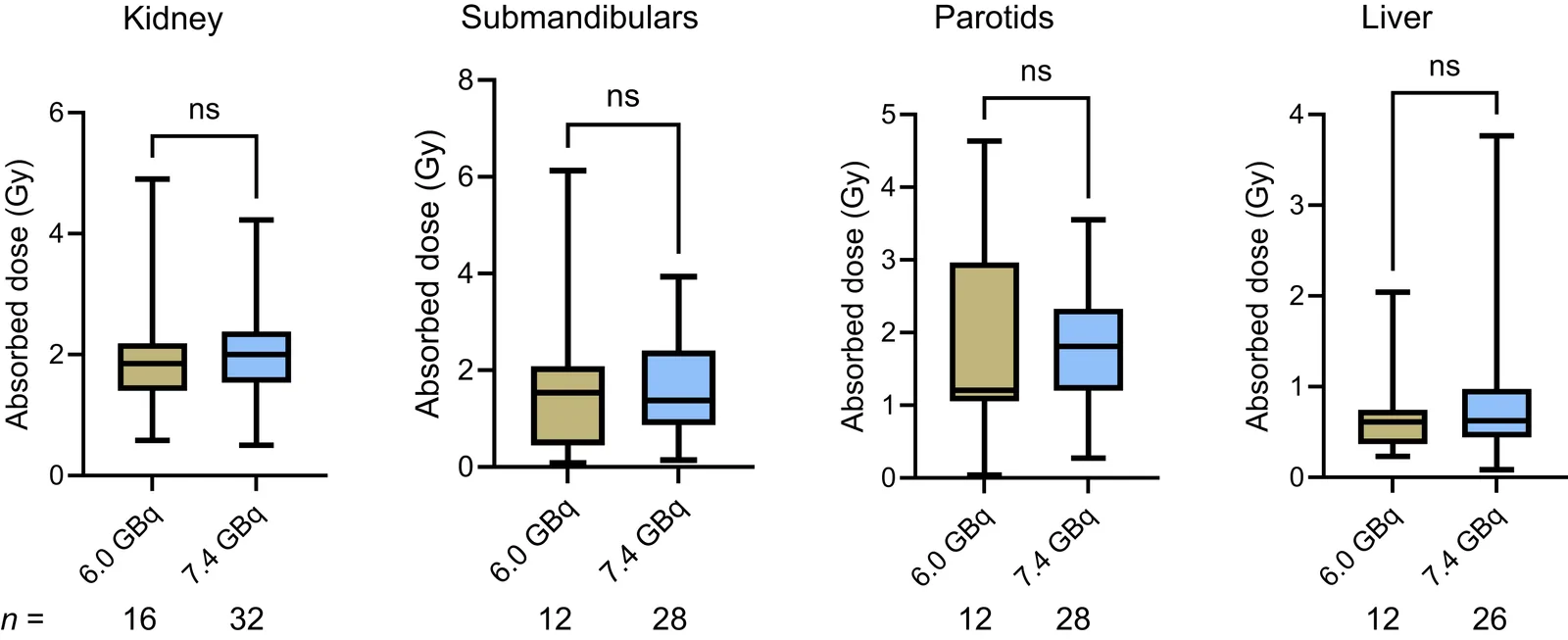
AD estimates in organs at risk.

**TABLE 1.** Organ AD Estimates Over Whole Cohort (WC) and Between the 2 Different Injected Activity Cohorts

			AD		
Organ	Cohort	*n*	Gy	Gy/GBq	*P*
Kidney	WC	48	2.01 ± 0.81	0.29 ± 0.11	0.41
	6.0 GBq	16	1.94 ± 0.94	0.32 ± 0.14	
	7.4 GBq	32	2.05 ± 0.73	0.28 ± 0.09	
Submandibular glands	WC	40	1.59 ± 1.17	0.23 ± 0.18	0.99
	6.0 GBq	12	1.74 ± 1.59	0.29 ± 0.26	
	7.4 GBq	28	1.53 ± 0.91	0.20 ± 0.12	
Parotid glands	WC	40	1.79 ± 0.96	0.26 ± 0.15	0.63
	6.0 GBq	12	1.81 ± 1.34	0.30 ± 0.22	
	7.4 GBq	28	1.78 ± 0.74	0.24 ± 0.10	
Liver	WC	38	0.77 ± 0.64	0.11 ± 0.09	0.57
	6.0 GBq	12	0.68 ± 0.45	0.11 ± 0.08	
	7.4 GBq	26	0.82 ± 0.70	0.11 ± 0.09	

A theoretic extrapolation of the single-cycle renal AD to 6 total cycles (assuming standard-of-care–approved number of cycles) is shown in Supplemental Figure 2. Although this hypothetical extrapolation assumes identical kidney AD between cycles, it suggests that 1 patient from each treatment arm could have been identified as at risk for exceeding 23 Gy to the kidneys by cycle 6. Neither patient developed nephrotoxicity based on the entire treatment course.

### Tumor Dosimetry

In total, 358 individual lesions from 45 patients were included in the analysis. There were 3 patients (6%) with no visible lesions by SPECT/CT who were excluded from analysis; therefore, the median number of lesions present in each patient was 7 (range, 0–18).

Figure 5 and Table 2 summarize the ADs between the 2 injected activities across the total tumor burden (for the selected lesions meeting analysis inclusion criteria) and individual lesions. The selected, bone, and lymph node lesion volumes were not statistically different between the 2 injection activity regimens of 6.0 and 7.4 GBq: median, 50.64 versus 63.32 (*P* = 0.716), 4.97 versus 5.92 (*P* = 0.557), and 4.14 versus 3.97 (*P* = 0.245) mL, respectively (Table 2). Similarly, the AD across the selected lesions were comparable between cohorts: median, 1.60 and 0.99 Gy/GBq (*P* = 0.5653) for the 6.0- versus 7.4-GBq groups, respectively. The mean AD was 1.74 ± 1.80 Gy/GBq to bone lesions (*n* = 304), 3.21 ± 3.40 Gy/GBq to lymph node lesions (*n* = 35), and 1.59 ± 1.45 Gy/GBq to liver lesions (*n* = 19). The only statistically significant difference in AD between the 2 injected activity regimens was observed in lymph node lesions, in which the cohort receiving 6.0 GBq demonstrated a higher reported AD (*P* < 0.001). Figure 6 depicts the AD to individual lesions across all patients, highlighting the variability in lesion AD at the inter- and intrapatient level.

**FIGURE 5.**
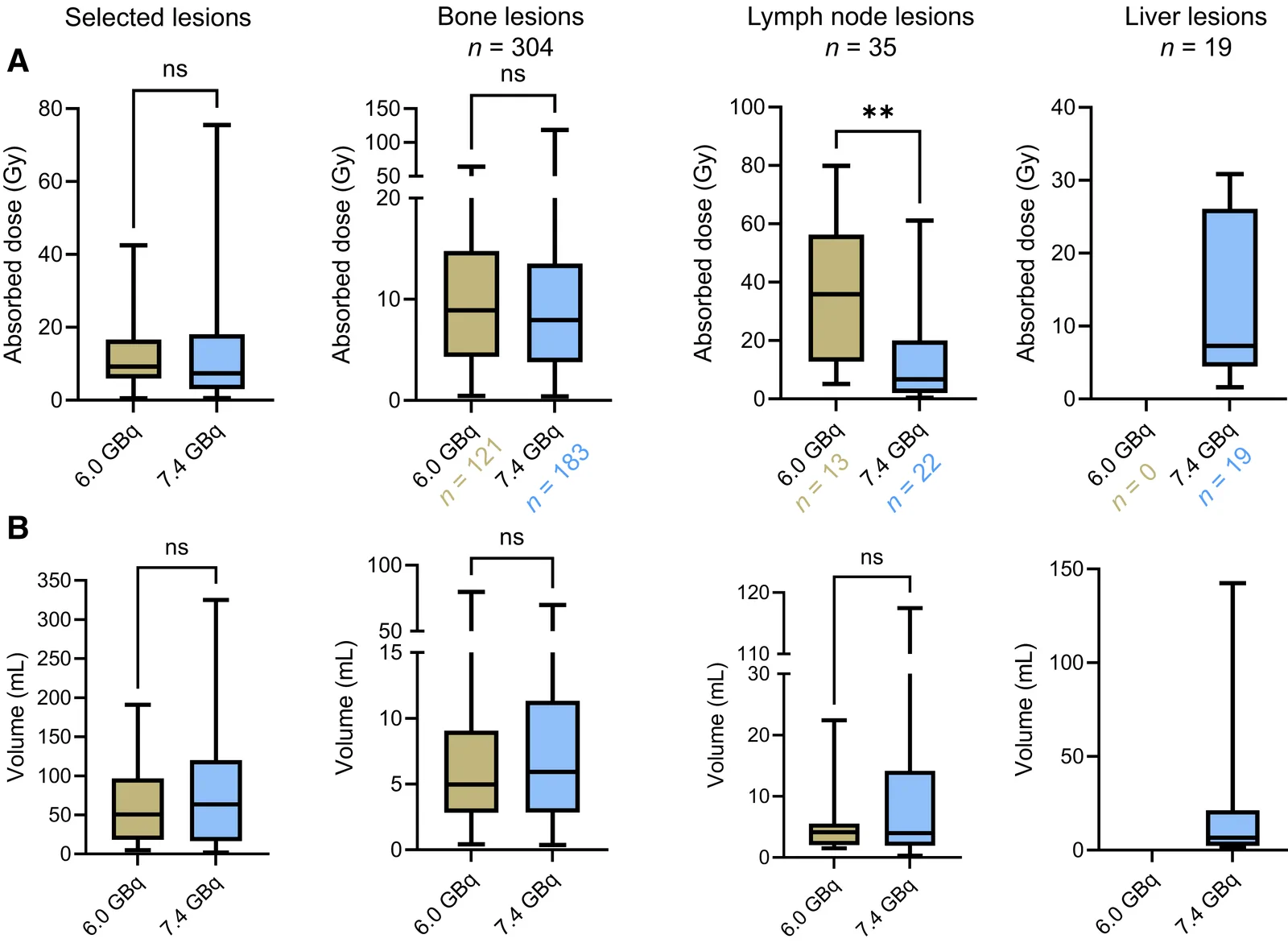
Lesion characteristics. (A) ADs. (B) CT-based lesion volumes included in AD estimate. Of note, only statistically significant value was in lymph node lesions AD (*P* < 0.001). ns = not significant.

**TABLE 2.** Lesion AD Estimates Over the Whole Cohort (WC) and Between the 2 Different Injected Activity Cohorts

				AD		
Organ	Cohort	*n*	Volume (mL)	Gy	Gy/GBq	*P*
Total tumor burden	WC	45	78.05 ± 77.81	12.74 ± 13.96	1.84 ± 1.95	0.565
	6.0 GBq	16	62.90 ± 49.40	12.30 ± 10.44	2.05 ± 1.74	
	7.4 GBq	29	86.41 ± 87.43	12.73 ± 14.87	1.72 ± 2.01	
Bone lesions	WC	304	—	11.95 ± 12.61	1.74 ± 1.80	0.533
	6.0 GBq	121	7.75 ± 9.48	11.45 ± 11.38	1.90 ± 1.83	
	7.4 GBq	183	9.37 ± 10.26	12.27 ± 13.35	1.64 ± 1.77	
Lymph node lesions	WC	35	—	21.14 ± 21.13	3.21 ± 3.40	0.001
	6.0 GBq	13	5.32 ± 5.21	34.39 ± 23.90	5.61 ± 3.93	
	7.4 GBq	22	16.35 ± 30.26	13.31 ± 14.42	1.79 ± 1.96	
Liver lesions	WC	—	—	—	—	
	6.0 GBq	0	—	—	—	
	7.4 GBq	19	19.85 ± 34.02	11.87 ± 10.62	1.59 ± 1.45	

**FIGURE 6.**
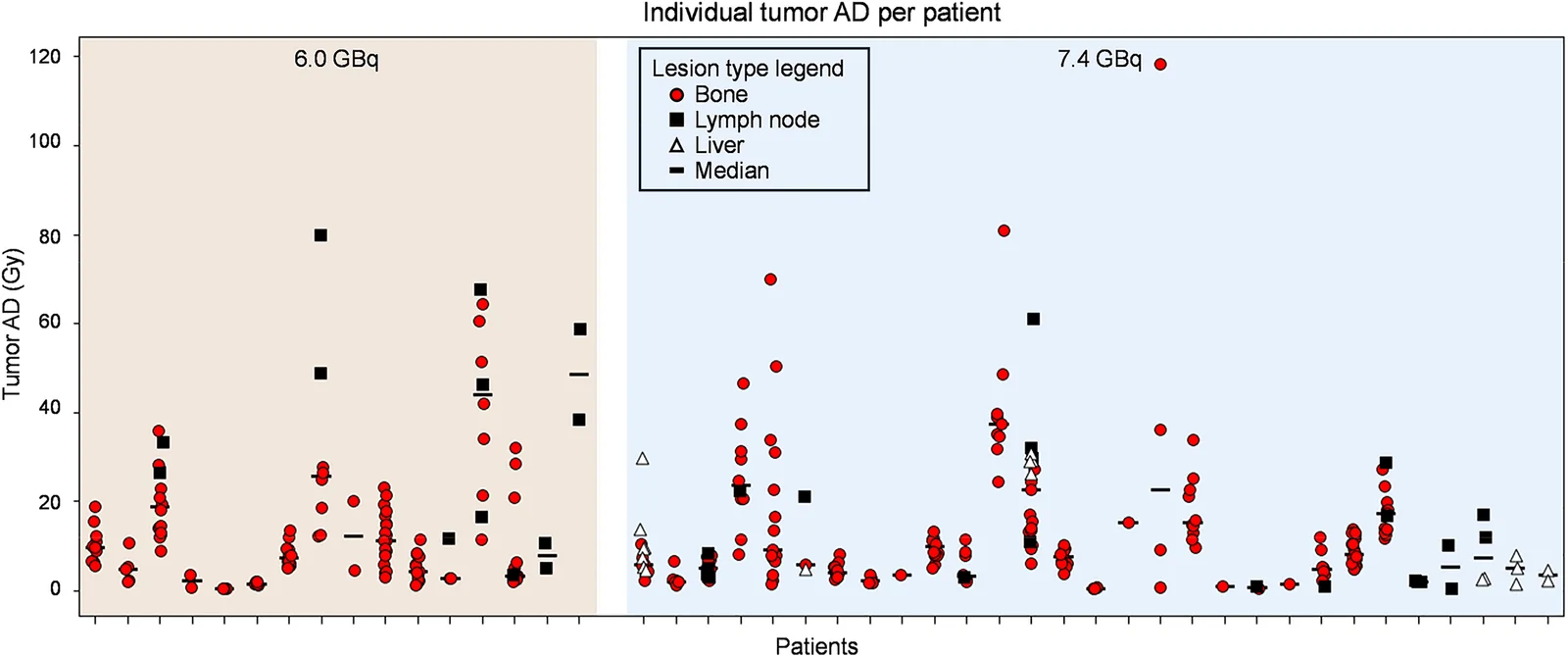
Variability in calculated lesions ADs based on injected activity and anatomic location on and inter- and intrapatient level.

### Clinical Outcomes

In the 6.0- and 7.4-GBq groups, 8 of 16 (50%) and 16 of 32 (50%) patients completed all 4 cycles, with 7 of 16 (44%) and 9 of 32 (28%) achieving a PSA decline greater than 50, respectively. Median OS between the 6.0- and 7.4-GBq arms was found using the Mantel–Cox test: 467 versus 420 d (*P* = 0.3923) (Supplemental Fig. 1).

The median PSA-PFS and OS across the whole cohort of the 48 included patients were 4 mo (interquartile range [IQR], 3–8 mo) and 14 mo (IQR, 8–24 mo), respectively. The median PSA-PFS and OS for the 2 cohorts, 6.0 versus 7.4 GBq, were 3 mo (IQR, 3–9 mo) and 15 mo (IQR, 9–21 mo) versus 4 (IQR, 3–8 mo) and 13 mo (IQR, 8–29 mo], respectively. There was no significant difference between the 6.0- and 7.4-GBq arms for PSA-PFS, PSA doubling time, or OS. Summarized efficacy results can be found in Supplemental Table 1.

Adverse events are summarized in Supplemental Table 2. Of 48 patients, 42 (88%) reported at least 2 adverse events; 19 of 48 patients (40%) reported mild adverse events, primarily digestive (e.g., nausea, vomiting, diarrhea), fatigue, pain, or dry mouth. Only 2 serious clinically determined adverse events were determined in 2 patients; thrombocytopenia (45 d after therapy) based on blood test and pleural effusion (4 d after therapy) defined by CT.

### AD Correlation with Clinical Outcomes

Correlation of the ADs with adverse events, efficacy, and quantitative clinical parameters derived in this analysis can be found in Figure 7. Of note, there were only 6 (3/6 from the 6.0-GBq group) and 3 (1/3 from 6.0-GBq group) adverse events reported for bone marrow– and kidney-related events, respectively, thereby limiting correlation analyses of sample sizes for organ AD outcomes.

**FIGURE 7.**
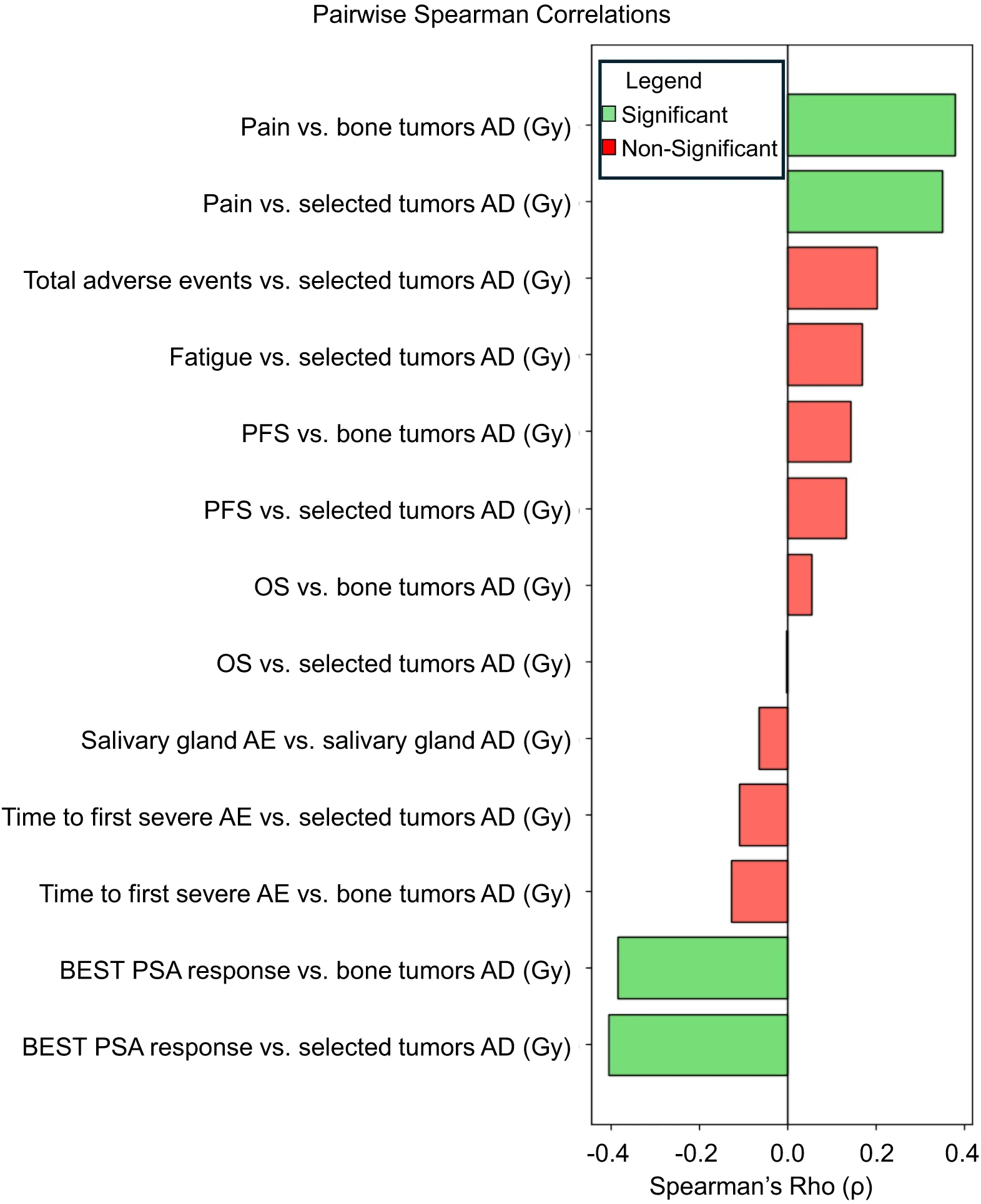
Correlation analysis between adverse events and quantitative parameters. Selected tumor AD represents total tumor burden that met inclusion criteria. “Total adverse events” represents all adverse events that were reported. AE = adverse event.

No significant correlation was found between tumor ADs and survival outcomes for either bone tumors (OS, ρ = 0.053, *P* = 0.753; PFS, ρ = 0.142, *P* = 0.394) nor the total selected tumor burden (OS, ρ = −0.004, *P* = 0.982; PFS, ρ = 0.132, *P* = 0.389). However, pain scores showed a positive correlation with both bone tumor AD (ρ = 0.378, *P* = 0.018) and selected tumor AD (ρ = 0.350, *P* = 0.019), both reaching statistical significance. Best PSA response showed moderate negative correlations with statistical significance for both bone tumor AD (ρ = –0.384, *P* = 0.017) and selected tumor AD (ρ = –0.404, *P* = 0.006), indicating that higher tumor AD was associated with deeper PSA declines. In contrast, no significant correlations were observed between selected tumor AD and total adverse events (ρ = 0.201, *P* = 0.185), fatigue (ρ = 0.169, *P* = 0.266), or time to severe adverse event (ρ = –0.110, *P* = 0.472). The same held true between bone tumor AD and time to severe adverse event (ρ = –0.128, *P* = 0.444). Salivary gland dose was not significantly correlated with salivary gland adverse events [36 of 48 patients (75%) experienced an adverse event] (ρ = –0.065, *P* = 0.695).

## DISCUSSION

In this analysis of 48 patients with mCRPC from RESIST-PC, a randomized, prospective, phase 2 trial, the ADs of [^177^Lu]Lu-PSMA-617 based on a hybrid imaging protocol (planar + SPECT) were not different between the 6.0- or 7.4-GBq injected activity arms. To our knowledge, at the time of publication, this study constitutes the largest prospective trial dataset examining the AD of [^177^Lu]Lu-PSMA-617 to organs at risk and tumors and their relationships with safety and efficacy.

Direct comparisons of this work with other published dosimetry studies are challenged by differences in dosimetry image acquisition, reconstruction parameters, lesion segmentation approaches, AD computation methodology, and applied correction factors. Nevertheless, prior retrospective hybrid imaging dosimetry studies with larger cohorts report higher kidney ADs as high as 2-fold greater compared with those in this study (18,19). Furthermore, the organ AD results published here are slightly lower than those of the VISION clinical trial (20), perhaps due to methodology here where we omit overlapping organ activity in the AD estimate. The SPLASH clinical trial (notably evaluating a different tracer, [^177^Lu]Lu-PSMA I&T) reported a lower tumor-to-kidney AD (21) ratio than we found here, consistent with a prior report (22). The estimated ADs from the 2 activity regimens (6.0 and 7.4 GBq) were comparable in both organs at risk and tumors included in the analysis.

Radiopharmaceutical AD thresholds for adverse event toxicity used by regulatory agencies are evolving but have been historically derived from experience with external beam radiation therapy. For the kidneys, the tolerance dose corresponding to a 5% risk of nephrotoxicity at 5 y has been estimated at approximately 18–23 Gy for bilateral whole-kidney irradiation (23). In our dataset, hypothetically extrapolating the kidney AD from a single cycle up to the clinically approved 6 cycles, assuming consistent cycle doses, resulted in only 2 patients exceeding 23 Gy (Supplemental Fig. 2), neither of whom developed nephrotoxicity.

Of note, the hybrid imaging dosimetry protocol used in this study has limitations. In this approach, the planar scans provide the shape of the time–activity curves, whereas the single quantitative SPECT/CT provides an accurate activity measurement allowing for a scaling factor to be implemented (24). Although planar imaging has historically had a role in dosimetry assessments, the susceptibility to activity overestimation (25–27) and region overlap limits the accuracy of AD estimates. As SPECT acquisition times continue to improve, the traditional tradeoff between the speed and ease of planar imaging and the superior quantitative accuracy of 3-dimensional imaging is becoming increasingly negligible. Of note, the late imaging time point obtained in 7 patients at more than 168 h postinjection provided valuable information regarding biodistribution, particularly for lesion time–activity curve generation.

A major limitation of this study was the absence of bone marrow dosimetry and the limited accuracy of salivary gland AD assessments due to the imaging protocol. Although bone marrow uptake surrogates, such as lumbar vertebrae, can be incorporated into imaging-based dosimetry assessments, bone marrow AD was not feasible in this study because of overlapping activity with skeletal disease burden (28). The inability to reliably estimate bone marrow AD is particularly significant given the risk of hematoxicities. Salivary gland dosimetry in this study was influenced heavily by patient head movement during acquisitions, causing difficulties in region propagation and activity spillover. Other studies have reported that CT-based anatomic segmentations can underestimate functional uptake volumes (17).

Given the imaging quantification limitations of planar imaging, it is our recommendation that future dosimetry studies be designed using multiple-time-point SPECT/CT imaging protocols rather than the hybrid approach investigated in this work.

Clinically, outcomes were comparable between patients receiving 6.0 or 7.4 GBq of [^177^Lu]Lu-PSMA-617, with similar PSA-PFS and OS (Supplemental Table 1). Notably, the best PSA response correlated with higher tumor AD (Fig. 7). Although lymph node lesions in the 6.0-GBq group demonstrated ADs that were statistically higher than those in the 7.4-GBq group, this difference likely reflects lesion inclusion bias and the relatively small sample size (*n* = 20).

The most frequently reported adverse events were gastrointestinal symptoms (nausea, vomiting, and diarrhea), as well as dry mouth, fatigue, and pain. As shown in Figure 2, delayed exposure throughout the gastrointestinal tract is visible on late imaging time points. A subanalysis is currently under way to evaluate the relationship between gastrointestinal AD and these adverse events.

Modulation of injected activity is of high interest; increased activities may enhance tumor AD and therapeutic response, whereas decreased activities can spare radiation exposure to off-target tissue. Higher injected activities exceeding the standard 7.4 GBq have already been safely reported (29).

In this work, injected activities of 6.0 GBq resulted in negligible differences in organ and tumor ADs compared with 7.4 GBq, with no difference in adverse event incidence. The observed similarities in AD/GBq between cohorts supports an expected linear scaling of AD within this activity range. However, AD is also governed by the dynamic cellular targeting environment, including possible target saturation, radiopharmaceutical redistribution, or nonlinear pharmacokinetics, none of which was evaluable in this dataset but remains a key question in the field with increased injected activities.

A limitation of this analysis is that comparisons were performed between patient groups randomized to different administered activity regimens, rather than within the same lesions in the same patient. Consequently, differences in AD and outcomes may partly reflect biologic heterogeneity, including variations in biodistribution, target expression, lesion size, and intrinsic radiosensitivity. The AD equivalence between treatment groups may be explained by other heterogeneous microenvironmental and histopathologic factors (e.g., perfusion variability, receptor density heterogeneity, necrosis, fibrosis, interstitial transport limitations, etc.). Although the large number of analyzed lesions enabled robust statistical analyses and highlighted substantial variability in tumor ADs, caution is warranted when interpreting dose equivalence across groups. Nevertheless, the association between higher tumor AD and deeper PSA declines supports the biologic relevance of lesion-level dosimetry for treatment optimization.

## CONCLUSION

In this analysis of 48 patients with mCRPC who were treated with [^177^Lu]Lu-PSMA-617 in the RESIST-PC phase 2 trial, there were no differences in the estimated AD to the normal organs at risk and selected lesions between the 2 different injected activities groups (6.0 and 7.4 GBq). The increased AD was strongly correlated with a PSA decrease but an increase in patient pain. There was significant inter- and intrapatient variability in tumor ADs. The hybrid imaging approach is not a recommended methodology.

## DISCLOSURE

No financial relationships, commercial affiliations, or personal associations influenced the work reported in this study. All relationships below for each author are outside the submitted work. Wolfgang Fendler reports fees from SOFIE Biosciences (research funding), Janssen/JJ (consultant, speaker), Perceptive (consultant, image review), Bayer (consultant, speaker, research funding), Novartis (speaker, consultant, research funding), Telix (speaker, research funding), GE HealthCare (speaker, consultant), Eczacıbaşı Monrol (speaker), Abx (speaker), Amgen (speaker, research funding), Urotrials (speaker), Lilly (consultant), AstraZeneca (research funding), Curium (consultant), and BMS (speaker), outside of the submitted work. Ken Herrmann reports fees from Advanced Accelerator Applications, a Novartis co., Amgen, AstraZeneca, Bain Capital, Bayer, Boston Scientific, Convergent, Curium, Debiopharm, EcoR1, Immedica, Isotopen Technologien München, Janssen, Merck, Molecular Partners, POINT Biopharma, Pfizer, Radiopharm Theranostics, Rhine Pharma, Siemens Healthineers, Telix, Theragnostics, Ymabs, Fusion (board advisory), GE HealthCare (board advisory), Novartis (grants, IIT funding), SOFIE Biosciences (fees, stock, grants, IIT funding), Pharma15 (stock), NVision (stock, fees), Convergent (stock), Aktis Oncology (stock), and AdvanCell (stock), all outside of the submitted work. Jeremie Calais reports grants from support to his institution from Lantheus, Novartis, and POINT Biopharma. He also reports consulting activities (advisory board member, speaker, blinded reader) for Advanced Accelerator Applications, Amgen, Astellas, Bayer, Blue Earth Diagnostics Inc., Curium Pharma, DS Pharma, Fibrogen, GE HealthCare, Isoray, IBA RadioPharma, Janssen Pharmaceuticals, Monrol, Lightpoint Medical, Lantheus, Novartis, Pfizer, POINT Biopharma, Progenics, Radiomedix, Sanofi, Siemens-Varian, SOFIE, and Telix Pharmaceuticals, outside the submitted work. Matthias Eiber reports fees from Blue Earth Diagnostics Ltd. (consultant, research funding), Novartis/AAA (consultant, speaker), Telix (consultant), Bayer (consultant, research funding), RayzeBio (consultant), POINT Biopharma (consultant), Eckert-Ziegler (speaker), Urotrials (speaker), Janssen Pharmaceuticals (consultant, speakers bureau), Parexel (image review), and Bioclinica (image review), outside the submitted work, and a patent application for rhPSMA. He and other inventors are entitled to royalties on sales of POSLUMA. Matthias Eiber serves on advisory boards or steering committees for Novartis, Blue Earth Diagnostics, and Telix. Machael Lassmann reports Institutional grants from PentixaPharm. Johannes Czernin is a scientific advisor and founder of SOFIE Biosciences and Infinity Topco; founder of Trethera Therapeutics; and scientific advisor for Aktis Oncology. No other potential conflict of interest relevant to this article was reported.

## Notes

KEY POINTS **QUESTION:** Within our patient cohort of 48 patients with mCRPC who were treated with [^177^Lu]Lu-PSMA-617 in the RESIST-PC phase 2 trial, are ADs in organs at risk and lesions affected by the 2 injected activity regimens (6.0 and 7.4 GBq), and how are they associated with treatment response and adverse events? **PERTINENT FINDINGS:** The hybrid imaging approach is not a recommended methodology for AD estimation [e.g., organs-at-risk limitations (omitting bone marrow), planar activity overlap per region, and time to complete computation]. The organs-at-risk ADs calculated here were less than those published in the VISION trial, perhaps due to methodology of compensating for activity overlap per region; for lesions, there was significant inter- and intrapatient variability in tumor ADs. **IMPLICATIONS FOR PATIENT CARE:** There was a strong correlation found between the AD and PSA decrease, at the expense of increased patient pain.
